# Prison Training and Administration

**Published:** 1898-03-12

**Authors:** 


### Modern Sociology.

### PRISON TRAINING AND ADMINISTRATION.

The report of the Prison Commissioners, lately issued, bears emphatic testimony to the fact that they are absolutely determined to render the working and administration of our English prisons as perfect as possible, and to this end they are introducing many improvements which we shall be glad to have an opportunity of bringing before our readers. The subject is one of great importance to all interested in the criminal classes; and as we can supplement the official reports by practical experience of the interior life of prisons, we trust to be able to show what may be done in the future, as well as what has already been accomplished in the past.

The secretary of the Howard Association, Mr. Tallack, in a recent letter to the *Times*, enters on a brief account of a system lately inaugurated for the training of prison warders, both male and female. This is a very important department, although, as we intend to show, it is not the only one in which improvements may be advantageously introduced. The secretary gives a short description of the training schools which have now been established for the practical education of warders—two for men at Chelmsford and Hull, one for women at Wormwood Scrubbs, and there is also another for them—which he does not mention—at Manchester. We shall hope later to give a full detail of the plans on which these schools are worked; but the subject is one of a very extensive range, and as it happens that we have been repeatedly asked to give advice in these pages to persons desirous of entering the prison service we shall, perhaps, deal with the matter most usefully if we begin by explaining the process from first to last, necessary to be employed by all who are anxious to devote themselves to that employment. Many of those who have applied to us for advice have been young women already trained as nurses, whose main desire is to be occupied in the care of sick prisoners, and we will, therefore, commence by explaining to such individuals the preliminary steps which must be taken in all cases by those aspiring to become female warders. They must understand that no application to enter the prison service simply as nurses would be entertained; they must begin as ordinary female warders, although the fact that they had learnt nursing would be favourable to their appointment, if in all other respects they were eligible. The young woman who desires to serve under the Prison Commissioners must be not under the age of 23, or over that of 40, and she must not be less than 5 ft. 2 in. in height; it will be an advantage to her if she is considerably over that stature.

Her first step must be to go to the governor of the prison nearest her own residence, and state to him that she wishes to enter the service. Having ascertained that in age and height she is suitable, and expects, also, to be able to bring a medical certificate of perfect soundness in body and mind, he will give her a printed paper, which she will have to fill up with a formal application for an appointment as warder. This is forwarded to the Prison Commissioners, and she awaits their reply. When it comes, if favourable so far, she will have to go back to the adjoining prison, there to be examined in reading and arithmetic, which are essentials as to education, though by no means all the requirements which it would be well for her to possess. There is no special limitation as to the religious profession, only all must alike attend the chapel services when on duty. If her testimonials on all these various points have proved satisfactory, and if she has also successfully "passed the doctor"—as the current phrase goes—she is informed that her application has been granted, a result which would certainly be rendered more likely, by her having been able to state that she possessed a trained knowledge of nursing. She must then wait for a vacancy to occur in some suitable prison. This will always, as a rule, be chosen for her in a large prison, and one at a distance from her home. Probably she will not have to wait more than two or three months at the longest. Then an appointment will be intimated to her, and her uniform will be provided; this consists of a black woollen dress, a small black bonnet, and a leather belt to which her keys are attached by a long and massive steel chain. As there are now the two recently established training schools for female warders at Wormwood Scrubbs and Manchester, it is possible that she may be sent to one of them before being definitely placed in the position of a warder. In that case she would not, while only a probationer in training, receive a uniform or be entrusted with the keys.

The arrangements for the board and lodging of probationers in the training schools, differ somewhat at present in the case of women from that which has been organised for the men; but only, probably, because the short period which has elapsed since the establishment of the training system has not given time for suitable accommodation to be provided for the female probationers. Both at Hull and Chelmsford, where the men are educated, they lodge and board within the prison walls, and each man has a bed-room to himself, four meals a day being provided for him, with also the use of a library, where newspapers as well as books are at his command. But the arrangements for women, with whose position we are dealing exclusively at present, have hitherto been different. A new block of quarters are being built at Wormwood Scrubbs, and until they are quite completed there could not be accommodation for the probationers within the walls. The governor of the prison has therefore hitherto settled with male married officers whose quarters are outside the building, to receive as lodgers for a term of about four months one or more of the female probationers, for whom a sufficient board is paid, although they receive no salary till regularly appointed on the completion of their training. During the day each probationer is placed under the charge of an experienced warder, who has the entire care of a ward or gallery. This officer, along with others in the different departments, instructs them in the whole working machinery of the prison. The head matron is, however, the person responsible for their training, and they pass a considerable time every day in her office learning the rules. We shall hope in our next issue to deal theoretically with these and kindred subjects.

